# Circles of Connection: Visualizing Human–Nature–Animal Bonds Through Participatory Art in Wildlife Tourism

**DOI:** 10.3390/ani16091376

**Published:** 2026-04-30

**Authors:** Yulei Guo, David Fennell

**Affiliations:** 1 Tourism Department, Chengdu Research Base of Giant Panda Breeding, Chengdu 610016, China; 2 The Conservation of Endangered Wildlife Key Laboratory of Sichuan Province, Chengdu 610016, China; 3 Tourism and Geography Department, Brock University, Catharines, ON L2S 3A1, Canada

**Keywords:** connection to nature index, visual participatory methods, giant panda, pets, nature-animal-human bond, conservation

## Abstract

Understanding human–nature–animal relationships is central to conservation and visitor management, yet these relationships are commonly studied through language-based surveys that may exclude participants across age groups and diverse cultural or educational backgrounds. This limitation highlights the need for more inclusive and experience-sensitive approaches capable of capturing relational meanings beyond verbal expression. This study adopts a visual participatory approach in which volunteer tourists were invited to draw circles representing themselves in relation to images of a giant panda, nature, and a pet. Extending the visual idea of Schultz’s Inclusion of Nature in Self in the Connection to Nature Index, more than 1000 tourists at the Chengdu Research Base of Giant Panda Breeding produced over 3000 drawings. These drawings were systematically coded along three dimensions—circle size, orientation, and spatial relationship—and analyzed using multinomial logistic regression and non-parametric tests. The results reveal consistent yet differentiated patterns of visual representation. Participants most frequently expressed relationships with nature and pets through enclosing circles, suggesting spatial inclusion, whereas relationships with the giant panda were more often represented through separate but proximal positioning, indicating a more mediated or observational mode of connection. Demographic factors, including age, residence, and visitation stage, significantly influenced drawing configurations, supporting the interpretation of connection as a context-sensitive and dynamic process rather than a fixed individual trait. Associations between drawing dimensions and self-reported pro-environmental orientation and momentary well-being were observed, although these relationships should be interpreted cautiously given the use of brief, context-specific indicators. Overall, the findings demonstrate that participatory drawing can function as both a research instrument and an engagement tool, enabling diverse visitor groups—including children—to express relational understandings of nature and wildlife. For conservation practice, such visual methods offer a scalable and low-barrier approach to visitor engagement, with potential applications in environmental education, interpretation design, and the assessment of human–animal relationships in situ.

## Simple Summary

Understanding how people relate to nature and animals is important for wildlife conservation and visitor management, yet many studies rely on written surveys that are not always accessible or engaging for all participants. This study explores a more inclusive approach using drawing. More than 1000 visitors to the Chengdu Research Base of Giant Panda Breeding were invited to draw simple circles to represent how they see themselves in relation to a giant panda, nature, and a pet. By analyzing the size and position of these circles, the study identified clear patterns. Participants most often represented their relationships with nature and pets through enclosing circles, suggesting a sense of closeness, while relationships with the giant panda were more often shown as separate but nearby. Age and background influenced how participants expressed these relationships, and some drawing patterns were associated with visitors’ self-reported feelings and environmental attitudes. These findings suggest that simple drawing activities can help people of different ages and backgrounds express their relationships with nature and animals. This approach offers a practical and engaging tool for conservation practitioners to better understand visitor experiences and design more inclusive communication and management strategies.

## 1. Introduction

Depictions of animals and humans represent some of the earliest forms of cave art in human history. From the cave paintings of Lascaux to contemporary visual culture, such representations have long served as a means of articulating relationships between humans and the more-than-human world [1]. Today, understanding the complex interconnections between humans, animals, and nature is increasingly urgent, as they underpin responses to global challenges such as biodiversity loss, habitat degradation, and ecological crisis.

In environmental psychology, this inquiry has been formalized through constructs such as the biophilia hypothesis [2], which posits an innate affinity for nature. Building on this, Mayer and Frantz [3] developed the Connection to Nature Index (CNI), a widely used instrument for assessing individuals’ emotional and experiential bonds with the natural world. The CNI and related measures have demonstrated strong predictive value for pro-environmental attitudes and subjective well-being [4], and have become central tools in studying human-nature relationships.

However, these instruments rely predominantly on language-based, self-reported metrics, which may not fully capture the affective and culturally situated dimensions of human-nature relationships [5]. While existing scales show strong internal consistency and convergent validity, they tend to privilege abstract cognition over alternative modes of expression [6]. In particular, the visual and spatial articulation of human connection to nature remains underexplored, despite its potential to reveal how individuals perceive relational proximity, significance, and identity in more intuitive and accessible ways [6].

To address this limitation, this study introduces a visual participatory approach that complements conventional survey instruments. Specifically, we combine the CNI with a structured drawing task in which participants represent their relationships with nature, pets, and giant pandas through spatial configurations of circles. This approach is conceptually informed by Schultz’s [7] Inclusion of Nature in Self (INS) scale, which employs overlapping circles to capture perceived interconnectedness between the self and nature. Unlike multi-item, cognitively oriented instruments such as the CNI, the INS emphasizes relational identity and psychological closeness through a simple visual metaphor [8,9]. Its pictorial format has proven particularly effective in capturing intuitive and holistic perceptions of human–nature relationships, especially among diverse participant groups, including children.

Building on this tradition, the present study extends the idea of visual-symbolic measurement by introducing a more flexible and expressive drawing task [10]. Rather than relying on a fixed diagram, participants actively construct their own spatial representations, allowing for variation in size [11,12,13], distance [9], and overlap [14]. These visual elements are interpreted as indicators of perceived relational proximity, salience, and centrality, drawing on prior research in visual cognition and self-representation. In this sense, the method does not seek to replace established scales but to complement them by capturing how individuals visually and spatially articulate their relationships with nature and animals.

Empirically, this study is situated at the Chengdu Research Base of Giant Panda Breeding (Panda Base), a major conservation tourism destination in China. As both a national icon and global conservation symbol, the giant panda provides a unique lens through which to examine human–animal–nature relationships in a real-world context. Drawing on a dataset of over 3000 drawings from 1188 participants, the study explores how visitors visually construct and differentiate their relationships with nature, pandas, and pets.

This study addresses two interrelated gaps. First, it advances CNI by integrating quantitative measures with structured visual participatory methods, enabling a more nuanced understanding of relational perception. Second, it examines how a charismatic species, the giant panda, mediates connections to nature within a high-profile tourism setting, contributing to ongoing discussions of affect, symbolism, and engagement in conservation contexts.

The study is guided by the following research questions:

RQ1: How do individuals visually represent their relationships with nature, pets, and giant pandas using spatial drawing elements (e.g., size, distance, overlap)?

RQ2: To what extent are these visual representations systematically associated across domains (nature, pets, and pandas)?

RQ3: How do visual representations relate to self-reported indicators of environmental attitudes and subjective experience?

By addressing these questions, the study contributes both methodologically—by expanding the use of visual participatory techniques—and practically, by informing more inclusive and emotionally resonant strategies for conservation communication and visitor engagement.

## 2. Literature Review

### 2.1. Connection to Nature Index

Studies employing the CNI are extensive, with numerous scales and variations developed over time. Whitburn, Linklater, and Abrahamse [15] illustrate that at least 17 different scales have been applied, expanding Mayer and Frantz’s [3] work into a 3-dimensional model: affect (feelings toward nature), cognition (knowledge and beliefs about nature), and behaviour (actions and experiences in nature). These scales, ranging from 1 to 40 items, are mainly self-report surveys requiring 5- to 7-point Likert scale responses. Tam [4] notes that, despite their variations, these scales are highly correlated and yield consistent results across these different tools. Gladys et al. [16], in a recent meta-analysis of 832 independent studies concerning connection to nature, concur that the conclusions of the studies, across geographical and cultural contexts, have been similar. It is widely believed that connecting to nature improves human cognition, physical and mental health, psychological well-being, and social skills [16]. However, despite their empirical robustness, these instruments are predominantly language-based and cognitively oriented, relying on abstract self-report measures that may not fully capture the affective, embodied, and contextually situated dimensions of human–nature relationships. North America (42%), Europe (28%) and Asia (25%) have been the most extensively researched regions; children’s (20%) voices are under-represented in past studies; and three studies employed pictorial contents [7,17,18].

In response to these limitations, visual approaches have been explored as complementary methods, although they remain relatively marginal within the field. One notable exception is Schultz’s [7] INS scale, which offers a more conceptual and symbolic approach to measurement. Adapted from Aron et al.’s [8,9] Inclusion of Other in the Self scale, the INS employs a single visual item—overlapping circles labeled “Self” and “Nature”—to represent perceived interconnectedness between the individual and the natural world. Rather than assessing discrete affective, cognitive, or behavioral dimensions, the INS draws on self-expansion theory and emphasizes relational identity and psychological closeness. As such, it captures a more intuitive and holistic sense of connection to nature and has proven effective across diverse participant groups.

**Figure 1:**
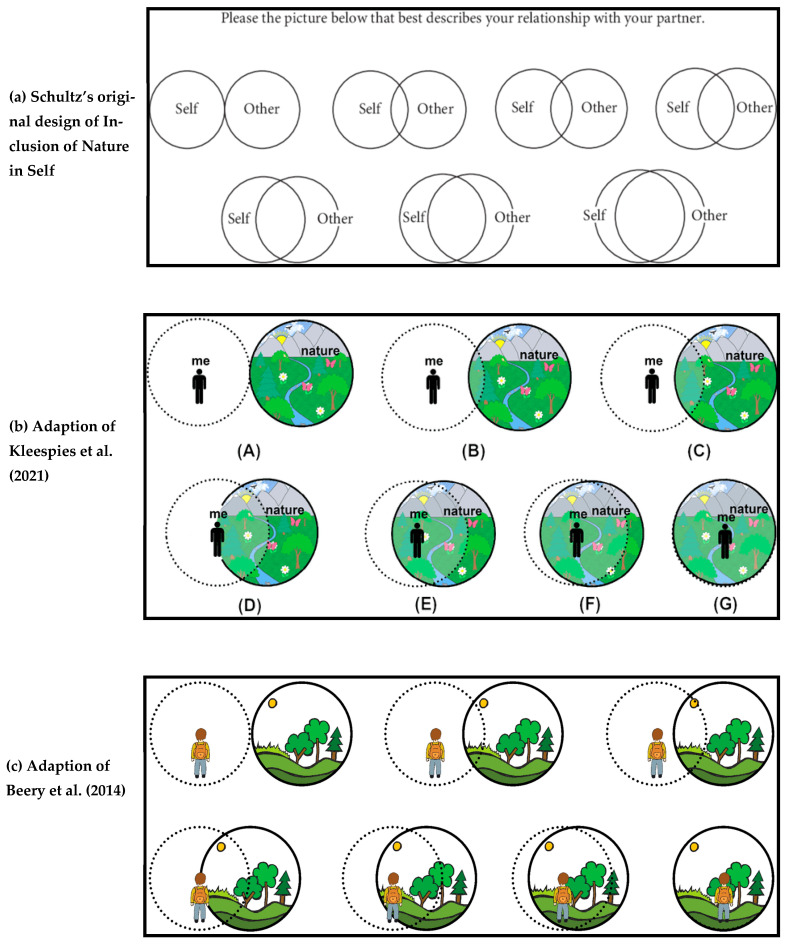
Schultz’s Inclusion of Nature in Self and more recent adaptations. (**a**) Schultz’s original design of inclusion of Nature in Self; (**b**) Adaption of Kleespies et al. [20]; (**c**) Adaption of Beery et al. [37].

The INS has been further applied and adapted in environmental psychology studies [12,19,20], in part due to its simplicity and visual format (Figure 1). Unlike conventional multi-item surveys, the single-item design is quick to administer and easy for participants to understand [10]. Its pictorial format also makes it especially accessible for children, who are often underserved in connection-to-nature research [21]. This is particularly relevant in light of findings from developmental psychology, which emphasize that children’s understanding of self and nature is formed through symbolic and often visual experiences [22,23].

In an effort to expand the theoretical and psychometric potential of the INS, Martin and Czellar [10] incorporated a free drawing task to explore how participants visually represent their self-nature relationships. Their findings demonstrated improved dimensionality, reliability, and validity, suggesting that graphic metaphors—such as spatial affinity [9], size [11,12,13], and centrality [14]—offer insights into the structure of nature connectedness. These developments point toward the potential of more flexible, participant-generated visual methods to capture relational aspects of human–nature connections that may not be readily accessible through standardized survey instruments.

### 2.2. Researching CNI with Tourists

Wildlife tourism provides a particularly relevant context for examining these methodological considerations. Researchers have recognized that wildlife tourism provides psychological and physical benefits to visitors who often connect with nature through experiences [24,25,26,27]. In more recent years, several empirical studies across have collected data showing that animal–human encounters experienced in wildlife tourism have a positive impact on visitors’ cognition, psychological well-being and overall health [28,29,30,31,32], further expanding Mayer and Frantz’s [3] three-dimensional CNI model.

However, similar to the broader literature, most studies in wildlife tourism rely heavily on text-based, Likert-type instruments and focus primarily on adult participants, leaving children and other less verbally oriented groups underrepresented [16]. In addition, cultural psychology research suggests that perceptions of the self–nature relationship vary across sociocultural contexts [33,34]. In collectivist societies, individuals may perceive themselves as more embedded within nature rather than separate from it, influencing how nature connectedness is experienced and expressed [35,36]. These insights further support the need for flexible and expressive measurement approaches.

The Panda Base, the largest ex situ conservation site for giant pandas, provides an ideal setting to explore these issues. It is also one of Chengdu’s most prominent tourism destinations. Previous survey-based studies conducted at the Panda Base, e.g., [38,39,40] have generated substantial data using QR-code-based questionnaires. Building on prior work in wildlife tourism research [38], simplified and visually oriented instruments such as the INS offer practical advantages in this setting. The one-item investigation approach reduces respondent burden, while pictorial representation enhances inclusivity for children and elderly visitors, who are often excluded from conventional survey research.

Furthermore, recent advances in conservation research emphasize participatory approaches that actively involve individuals as contributors to knowledge production [39,40]. Visual participatory methods, in particular, provide opportunities to capture more situated, expressive, and relational understandings of human–nature interactions. Building on these methodological and contextual considerations, the present study adopts and extends the visual logic of the INS by incorporating a structured drawing task. This approach enables participants to construct spatial representations of their relationships with nature and animals, thereby complementing conventional survey measures and addressing existing gaps in accessibility and expressive depth.

### 2.3. Spatial Metaphors: Animal and Nature

Animals are among one of the images [41,42,43,44] that human beings have marked on the walls of caves since the Upper Paleolithic age [45]. In a study of hybrid beings’ representations of animals and humans of this period, Dönmez [46] suggests that the conflict over power and possession has inspired these drawings, driving human beings to hide behind the images of powerful animals. Kalof [45] observes that animal images have been recruited purposefully in ancient Greek marble and stone sculptures, where “animals sleep alongside children, dogs gaze lovingly up at their human companions and birds and hares are carried about” (p. 22). Brady [47] concludes in her study of animals in contemporary environmental art that the animal–human relationship is about building up conflicting, harmonious, and in-between relationships in shared and daily spaces. These studies suggest how animals and humans have occupied visual space since the earliest human drawings, which have had significant symbolic implications for human society. For example, in Kalof’s [45] description, the side-by-side relationship between children and animals may imply a companionship between the two species, while the lower position the dog assigned by humans indicates domestication and ownership.

In his classic study of composition in the visual arts, Arnheim [48] writes that figures’ geometric aspects statically describe their size, location, orientation in space, and distance from one another. Even a blind or random production of an image, as Arnheim [48] puts it, carries a center as its “key point” (p. 1). Circles, combined with objects, can “give a center perceptual or intellectual definition by inference and construction” [1,48] Arnheim [48] believes that the animal–human relationship explored in visual compositions examines the conditions and functions of boundlessness and boundaries. Arnheim [48] reflects:

An animal painted on the wall of a prehistoric cave…as soon as art undertakes to show man in his world, it must show him in space; and to show him in space, a definite delimitation is almost indispensable. A frame of a particular size and shape defines the location of the things within its space and determines the distances between them (p. 43).

Arnheim’s words highlight that the animal–human relationship in visual spaces bears significance that can either make or break boundaries between two species. How human beings are positioned in the visual framework defines humans and animals in space and their contextual extensions. In this study, we propose that animal–human relationships can be explored and studied through their visual representations. More specifically, we focus on the iconic flagship animal, the giant panda, and domesticated pets, two species with whom contemporary human beings have fostered and formed significant contextual relationships.

## 3. Method

### 3.1. Site Selection

The Panda Base, located 10 km from Chengdu downtown, is the largest ex situ conservation site for giant pandas and a leading global institution for panda research. The site covers 3.07 km^2^ and housed more than 200 giant pandas during the study period. The Panda Base represents a highly intensive wildlife tourism setting in which human–animal and human–nature interactions are frequently enacted, observed, and socially mediated. Previous research has shown that visitor–panda relationships are dynamic and may vary across visitation frequency and stages of experience [49,50].

### 3.2. The Design of the Study

This study employed a cross-sectional field survey design combined with a visual elicitation task. Data were collected using a structured paper-based questionnaire administered on-site at the Panda Base.

The final survey of this study contained a total of 14 questions (including informed consent), organized into four sections.

Section 1 collected demographic information, including gender, age, and places of residence.

Section 2 examined visitation characteristics including first time visiting status, stage of visit (a. entering; b. at the enclosure; c. leaving).

Section 3 included three 5-point Likert-scale questions measuring visitors’ recycling attitudes, willingness to participate in giant panda conservation programs, and current self-reported happiness(“I am feeling happy now”).

These items were designed as brief, low-burden indicators of visitor behavioral orientation and in situ affective experience in a mixed-age wildlife tourism context, where survey length and cognitive demand must be minimized to ensure participation across children, adult visitors, and older participants. Specifically, the recycling attitude item was used as a contextual indicator of general environmental responsibility orientation in everyday public settings, rather than as a proxy for climate change mitigation behavior or comprehensive environmental ideology. The conservation participation item captured site-specific pro-conservation intention linked directly to panda protection activities, while the happiness item represented a single-item measure of immediate affective state, consistent with established practice in field-based tourism research where rapid assessment of visitor experience is required. Together, these variables function as supplementary contextual measures, providing behavioral and experiential background to complement the primary visual-elicitation data.

**Figure 2:**
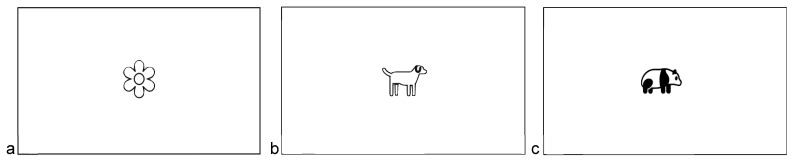
Drawing canvas for nature, pet, and giant panda. (**a**) A flower represents nature; (**b**) A dog represents pet; (**c**) A panda image represents panda.

The last and main section, printed on the back page of the questionnaire, had three visual participatory tasks (see Figure 2), which constituted the core methodological component of this study. Participants were presented with three pre-printed stimulus frames featuring a centrally placed image: (1) a flower (representing nature), (2) a dog (representing companion animals; only applicable to pet owners), and (3) a giant panda.

For each frame, participants were instructed to draw a circle representing themselves in relation to the stimulus. For example, in the flower condition, the instruction was:

“The flower represents nature. In this frame, please draw a circle representing yourself to describe your relationship with nature.”

This design was adapted from Schultz’s INS, but extended into a multi-stimulus visual format to capture relational positioning across different categories of human–nature and human–animal relationships.

Pre-printing the stimulus images at the center of each canvas standardized spatial reference points and reduced cognitive load during completion. Pilot testing indicated that free drawing of complex figures (e.g., drawing a panda) increased participant burden and reduced response willingness, particularly among children and older visitors. The simplified circle-based task was therefore adopted to improve accessibility and completion rates across demographic groups. Pet ownership was identified through a screening question, and only participants who reported owning pets completed the dog-related condition.

### 3.3. Field Implementation and Data Collection Procedure

Compared to QR-code-based digital surveys, the paper-based format was more easily accepted by visitors and reduced concerns related to digital privacy and link legitimacy commonly encountered in prior on-site research. Participation was particularly strong among older visitors and children, although young adults remained the dominant respondent group.

To address age-related sampling imbalance, targeted participation windows were implemented on selected field days, during which recruitment focused on children (<18 years) and older adults (>55 years). While this approach does not eliminate all self-selection effects, it improved demographic diversity and reduced reliance on digitally literate participants.

For child participants, data collection was conducted in the presence of parents or guardians, who assisted with reading and clarifying questionnaire items when necessary. The drawing task—the core component of the study—was completed directly by the children, following brief, standardized instructions provided by the researchers. The small-group, on-site survey format allowed researchers to observe the interaction process and ensure that children were able to engage meaningfully with the task. While parental assistance may have influenced some questionnaire responses, this procedure ensured accessibility while maintaining the integrity of the visual data produced by child participants.

Data were collected at two strategically selected locations to capture different phases of the visitor experience: the main entry/exit area and Giant Panda Villa No. 2, a high-attention enclosure site.

Each data collection session involved 4–6 participants at a time, allowing the researcher to provide instructions and supervise the visual task. This controlled group size was determined by spatial constraints of the field setting and the need to ensure standardized task administration. Participants received a small souvenir (a keychain or a panda magazine) upon completion as a token of appreciation. All completed paper questionnaires were subsequently digitized by a trained research assistant and entered into Wenjuanxing, from which structured Excel datasets were exported. All drawings were photographed and archived for subsequent coding and analysis.

### 3.4. Ethical Consideration

This study was conducted in accordance with the ethical principles outlined in the Declaration of Helsinki. Several measures were implemented to safeguard participants’ rights and privacy. First, all responses were collected anonymously; no identifying information was recorded, particularly in the paper-based survey, which ensured a higher degree of anonymity than typical online methods. Second, informed consent was obtained from all participants prior to data collection. For participants under the age of 18, informed consent was obtained from their parents or legal guardians prior to participation. Data collection was conducted in a public setting with voluntary participation and full anonymity, and no sensitive personal information was collected.

In line with international ethical standards, the study met the criteria for exemption from full ethical review, as it involved minimal risk and anonymized data. Moreover, according to Article 32 of the Notice on Issuing the Ethical Review Measures for Life Sciences and Medical Research Involving Humans (2023) issued by the Chinese government, studies that do not involve harm to participants, sensitive personal information, or commercial interests—particularly those using anonymized data or unobtrusive public observation—may be exempt from formal ethical review. Given that this study involved voluntary, anonymous participation in a public setting, with no collection of sensitive information, it qualified for exemption from institutional ethical approval.

### 3.5. The Coding Process

With more than 1000 visitors participating in this study and more than 3000 images produced, the coding task presented a challenge. Initially, we planned to identify the images through machine learning. However, the demands for high-quality images and visual standardization proved impossible for a small research team with limited resources like ours. Previous studies measured circles with rulers, e.g., [10,51], but the images studied were under 300. Instead, we adopted manual coding with more realistic and simplified goals. Building on these studies, three coding categories were developed to capture the information of the drawings, including the spatial relationship between the drawn circle and the printed images, the circle’s orientation, and the circle’s size.

**Figure 3:**
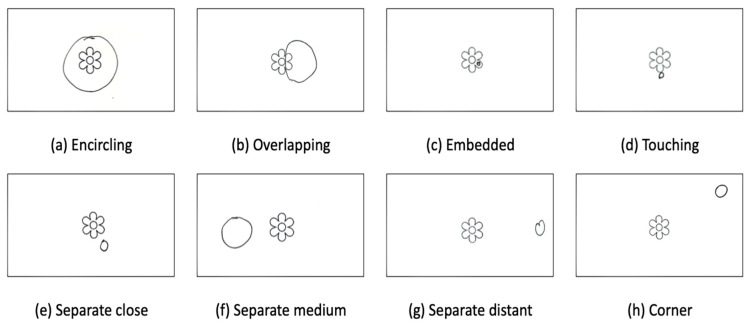
Examples of spatial relationships between Nature and the circle.

**Figure 4:**
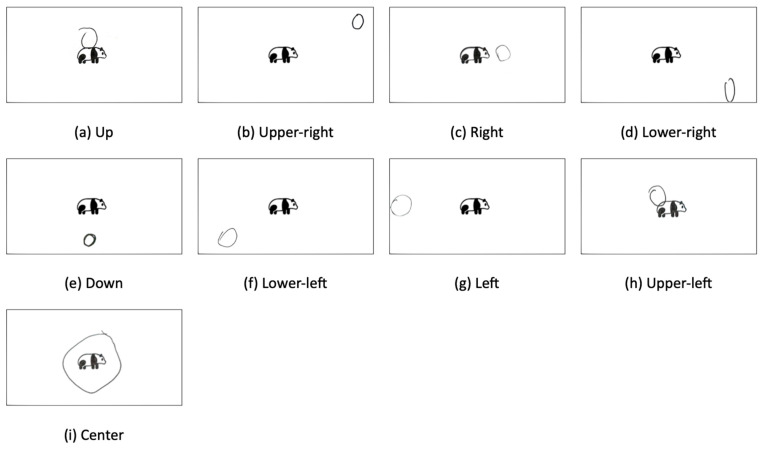
Examples of Orientations between Panda and the circle.

**Figure 5:**
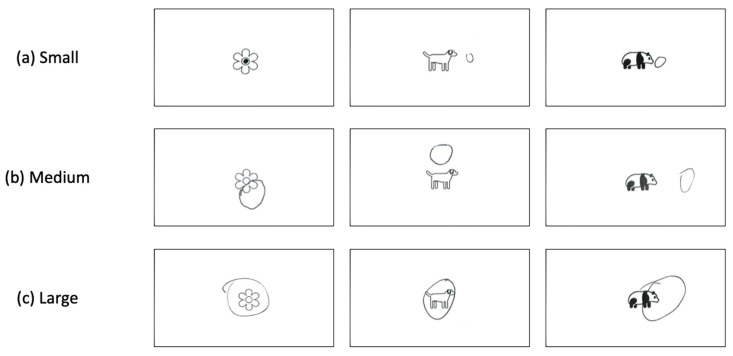
Examples of circle sizes for Nature, Pet, and Panda.

The spatial relationship describes how the circle has been placed in relation to the printed image, and the following categories were developed (see Figure 3. All images in Figure 3, Figure 4, and Figure 5 are reproductions of participant drawings). The same coding framework was applied consistently across the nature, pet, and giant panda conditions.

We developed nine categories capturing the orientations of the circle (See Figure 4). Three size-related categories were developed to capture the relative scale of the drawn circles (see Figure 5). To reduce subjectivity and ensure consistency, size classification was defined using a reference-based rule anchored to the boundary of the printed image. Specifically, a circle was coded as:

- Small if it could be fully contained within the boundary of the printed image;
- Medium if, when centered on the printed image, it intersected or extended beyond the image boundary;
- Large if it fully enclosed the printed image within the drawn circle.

This approach establishes a systematic visual reference point for all drawings, allowing size to be interpreted relationally rather than absolutely. While some drawings fell near category thresholds, borderline cases were resolved through comparison with the printed image boundary following the same rule-based criteria, ensuring consistency across coders.

A research assistant was employed full-time for one week to code the images. To ensure intercoder reliability and minimize subjectivity, a two-step procedure was implemented. First, a detailed codebook with visual examples and standardized definitions was developed by one of the lead researchers to guide the coding process. Second, the researcher independently coded a subset of 300 drawings (approximately 10% of the total sample) to serve as reference examples. She also provided training to the research assistant and supervised the entire coding process, reviewing and resolving any images flagged as ambiguous or irregular. Regular calibration meetings were held to refine category definitions and ensure ongoing consistency. This structured approach helped maintain coding reliability and allowed the team to apply the scheme consistently across the full dataset.

Quite a number of drawings were creative, offering words, extra images, multiple circles, and marks. These images are marked specifically, and a short description is provided for the creative inclusions. A few examples can be seen in Appendix A.

After cleaning up the dataset, 1188 entries were kept in the sample pool for further exploration. It is estimated that about 80 questionnaires were excluded from the paper document due to incomplete answers and unidentifiable drawings. The Cochran’s sample size formula suggests that, for a target population of 11 million (Visitor population of Panda Base in 2023), the minimum required sample size was 385 to ensure a confidence level of 95% and a margin of error of ±5%. The sample size of 1188 was chosen to ensure statistical reliability and robust representation of the target population. Compared to the QR code-based survey, the paper survey had a higher percentage of incompleteness. Also, one researcher spent about four hours verifying, quantifying, and organizing the data into a coherent set, an effort that was largely unnecessary in computer-based studies. A complete and cleaned dataset was then imported into SPSS 26.0 Mac. The three dimensions of the images were coded in SPSS for further statistical analysis (Table 1).

This study employed a combination of descriptive and inferential statistical techniques, including Chi-square tests with Cramér’s V, Kruskal–Wallis tests, Mann–Whitney U tests, and multinomial logistic regression (MNL). MNL was used to examine the extent to which demographic variables predict patterns in circle drawings. This approach is appropriate because the dependent variables—spatial relationship, orientation, and circle size—are categorical with more than two non-ordered outcomes [52]. Accordingly, MNL allows for the simultaneous comparison of multiple outcome categories without assuming ordinal structure. Effect sizes are reported as exponentiated coefficients (Exp β), interpreted as relative risk ratios. Values greater than 1 indicate an increased likelihood of a given outcome relative to the reference category, whereas values less than 1 indicate a decreased likelihood. Model fit was assessed using the likelihood ratio chi-square test and Nagelkerke’s pseudo R^2^. Alternative models, including ordinal and binary logistic regression, were considered but deemed unsuitable due to the non-ordinal nature of the dependent variables and the need to model multiple outcome categories concurrently. Therefore, MNL was selected as the most appropriate analytical approach for the data structure and research objectives.

## 4. Results

### 4.1. Sample Summary

Table 2 provides a summary of questions in Section 1 and Section 2. A relatively balanced age distribution was observed (Kurtosis = 0.558; SD = 14.912) with modest skew toward younger participants (Skewness = 0.801), suggesting that the paper-based design and targeted recruitment strategies were effective in engaging previously underrepresented groups. It is consistent with previous studies that women were more attracted to the survey. This gender imbalance may reflect both visitor composition at the Panda Base and differential engagement with the survey format, although these factors cannot be disentangled in the present study.

Table 3 indicates that participants reported strong support for pro-environmental activities such as recycling (Mean = 4.74) and giant panda conservation (Mean = 4.80), as well as a relatively high level of psychological well-being and reported happiness (Mean = 4.74). These high baseline values should be interpreted cautiously, as they may reflect social desirability or context effects in an on-site tourism setting.

### 4.2. Imagining Circles with Nature, Pets, and Pandas

**Figure 6:**
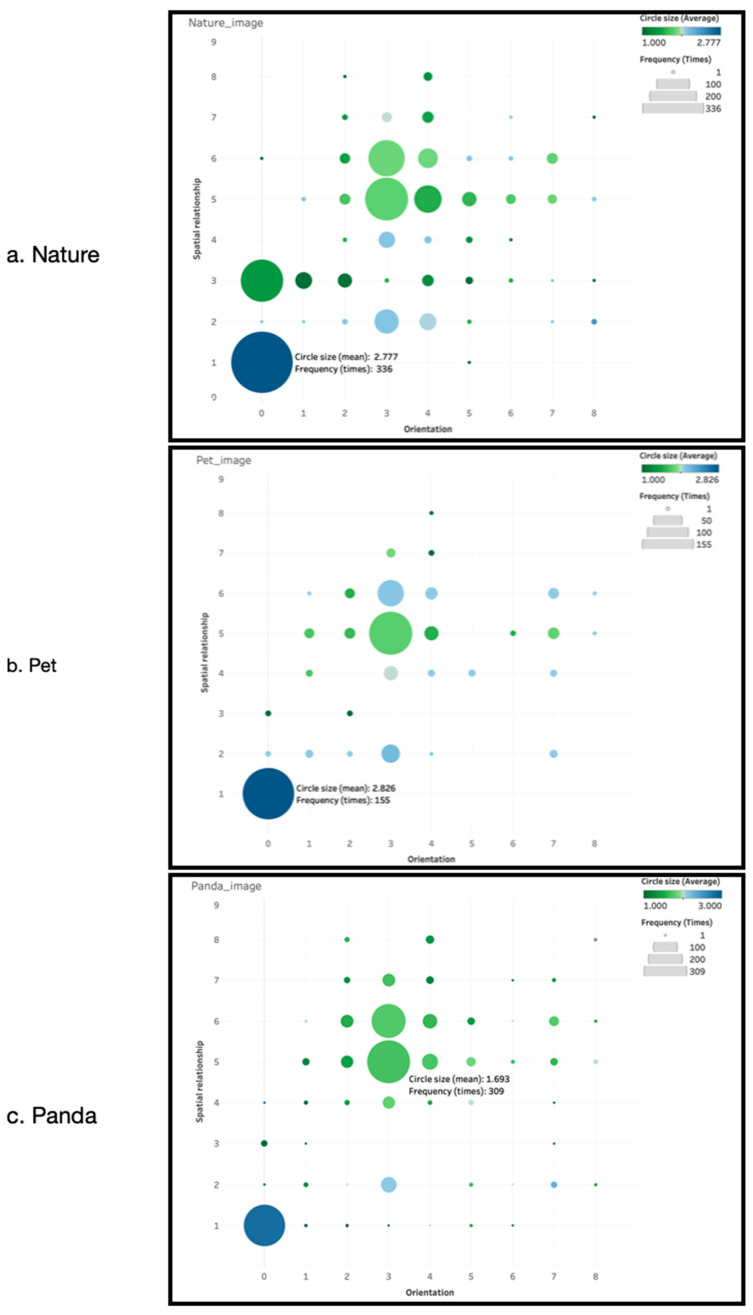
Spatial relationship, orientation, and circle size are visualized for Nature, Panda, and Pet.

The key research question guiding this study concerns how visitors visually differentiate their relationships with nature, pets, and giant pandas. Figure 6 provides a summary of visitor drawings in terms of the circles’ three dimensions. The y-axis represents the eight spatial relationship categories, and the x-axis represents the nine orientation categories. The distribution of points across the panel reflects the frequency of observed configurations, while point size indicates frequency, and color denotes the relative size of the drawn circles. Distinct patterns are observed across the three referents. Participants most frequently drew circles that encircle the images of nature and pets, reflecting a pattern of spatial inclusion. In contrast, drawings associated with the giant panda more commonly position the circle separate from, but in close proximity to, the printed image, suggesting a different spatial configuration.

**Figure 7:**
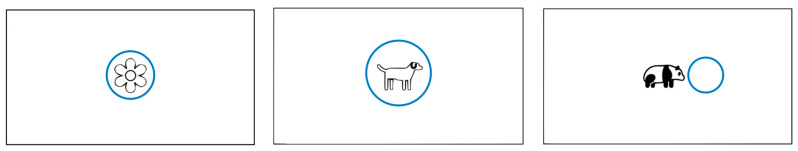
Visualizing the placements of circles for nature, pet, and panda.

Figure 7 further highlights the most frequent configurations. Encircling configurations dominate for nature and pets, with slightly larger circles observed for pets. For the giant panda, a medium-sized circle placed separately and closely on the right emerges as the dominant pattern.

Field observations indicated that participants sometimes associated encircling configurations with ideas such as care, protection, and closeness. In the case of pets, themes of responsibility and ownership were frequently mentioned, although these interpretations were not directly collected.

Participants more frequently described a close but separate relationship with the giant panda rather than an enclosing configuration. Importantly, these interpretations remain indicative rather than definitive, as the study does not directly measure participants’ intended meanings.

### 4.3. Demographic Factors and Their Influence on Circle Drawings

This section reports statistical associations between demographic variables and drawing characteristics. In this section, we present an analysis of images of nature and giant pandas, as only 433 pet owners contributed drawings of their pet. To ensure model stability and avoid overfitting, the suggested minimum of approximately 10 events per predictor variable was followed [53,54], resulting in the merging of a few categories in spatial relationship (e.g., the merge of “Corner” to “Separate distant”) and orientation (e.g., the merge of “Lower-left” and “Upper-left” to “Left”).

**Figure 8:**
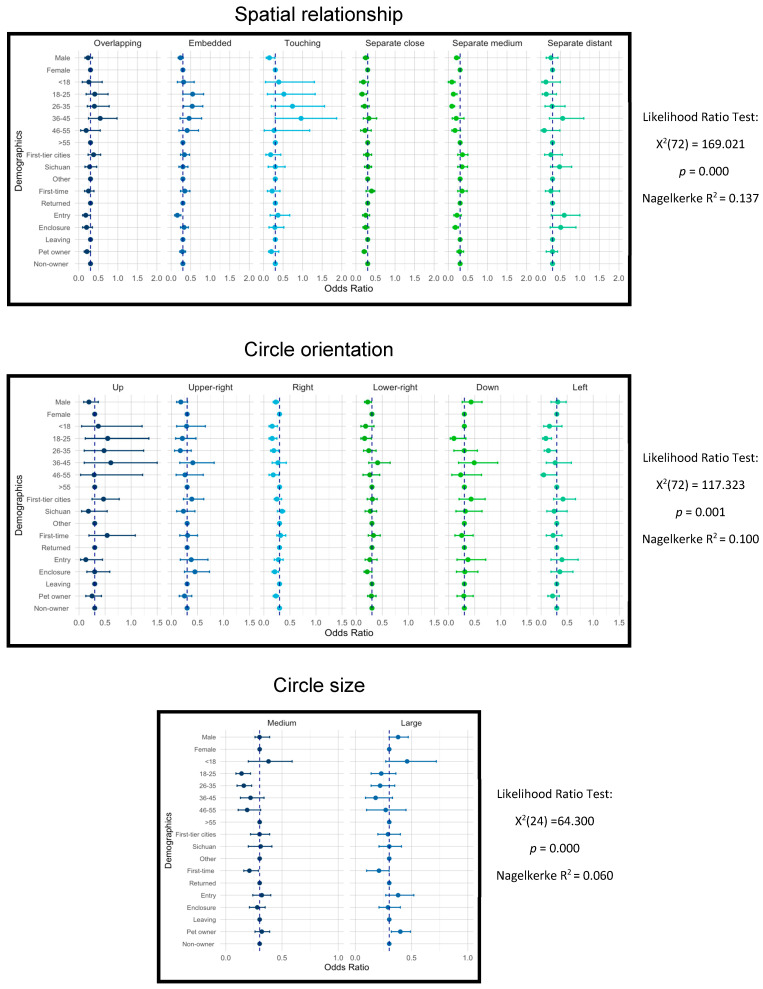
Plots of log transformed odds ratios and corresponding 95% credible intervals based on the multinomial logistic regression in NATURE circle drawings. The vertical dotted lines mark the odds ratio of 1.

Figure 8 demonstrates that in nature drawings, age, pet ownership, and visitation stage are significant predictors of spatial relationship patterns. For example, in age differences, for the 18–25 age group, embedded (Exp β = 2.52, *p* < 0.05) is significantly higher than for the >55 age group. In contrast, separate close (Exp β = 0.46, *p* < 0.05) and separate medium (Exp β = 0.35, *p* = 0.001) are significantly lower than the >55 age group. A similar tendency is observed in the 26–35 age group in the embedded (Exp β = 2.34, *p* < 0.05) and separate medium (Exp β = 0.24, *p* < 0.05) images. Visitation stage was also significantly associated with spatial configurations: participants at entry were less likely to produce embedded configurations (Exp β = 0.43, *p* < 0.05) compared to those exiting. Participants at the enclosures were less likely to draw separate medium circles (Exp β = 0.51, *p* < 0.05) than participants at the exit. For pet owners, overlapping (Exp β = 0.61, *p* < 0.05) and separate close (Exp β = 0.62, *p* < 0.05) have a lower likelihood than non-owners.

Also, age, visitation stage, and pet ownership are the most influential factors contributing to changes in the orientation of circles in Nature. Figure 8 demonstrates that the >55 age group showed a stronger tendency toward right-oriented placements, while the rest of the age groups demonstrate a significant departure from this pattern. Visitors at the giant panda enclosures tended to draw fewer circles on the right (Exp β = 0.63, *p* < 0.05) and lower right (Exp β = 0.64, *p* < 0.05) compared to visitors leaving the Panda Base. Participants without pets were much more likely to draw circles on the right than pet owners (Exp β = 0.69, *p* < 0.05).

Circle sizes on Nature were more significantly influenced by gender, age, and visiting frequency. For example, males had a 41% higher likelihood than females to draw large circles (Exp β = 1.41, *p* < 0.05). Participants in the 18–25 and 26–35 age groups had a 61% (Exp β = 0.39, *p* = 0.001) and 57% (Exp β = 0.43, *p* = 0.001) decrease in drawing medium-sized circles. First-time visitors were less likely to draw medium (Exp β = 0.63, *p* < 0.05) and large (Exp β = 0.63, *p* < 0.05) circles than returned visitors.

**Figure 9:**
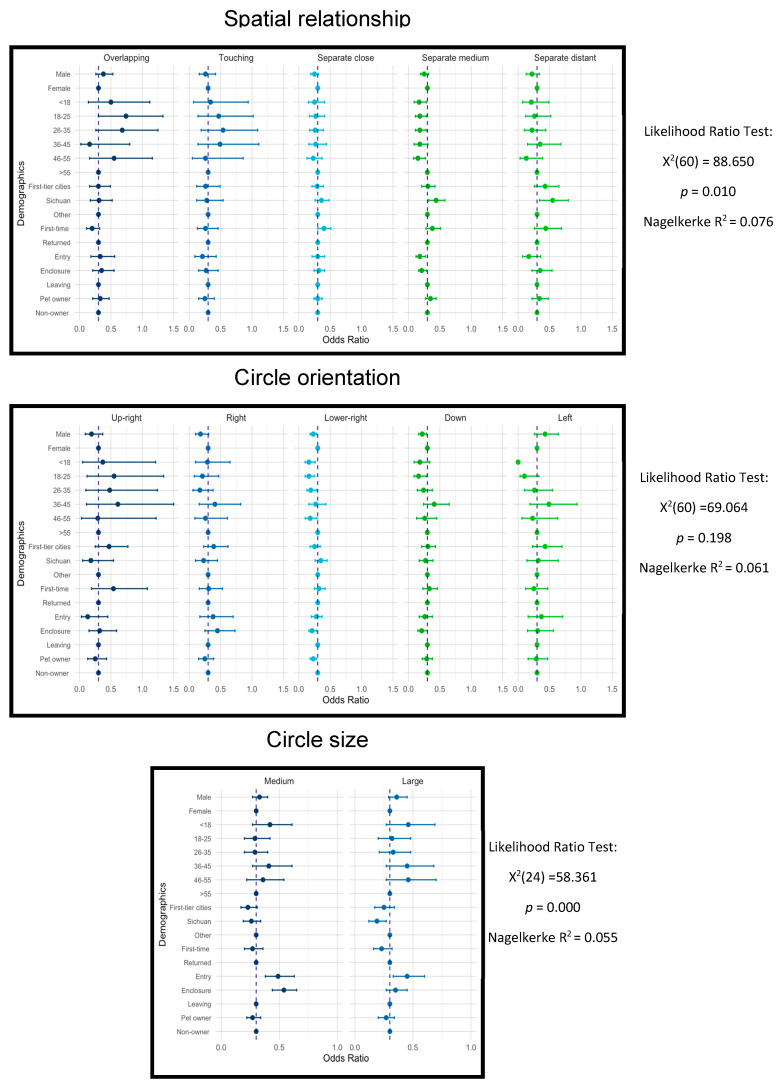
Plots of log transformed odds ratios and corresponding 95% credible intervals based on the multinomial logistic regression in PANDA circle drawings. The vertical dotted lines mark the odds ratio of 1.

Figure 9 shows how demographic factors have influenced circle drawings for the Panda image. The participants’ age, residence, and visitation stages significantly influenced the spatial relationship between circle drawings. Notably, senior participants (age > 55) were more likely to draw separate circles at a medium distance with the giant panda, while the rest of the age groups departed from it. Sichuan locals, compared to visitors from the first-tier cities and other regions, were 74% and 154% more likely to draw separate medium (Exp β = 1.74, *p* < 0.05) and distant (Exp β = 2.54, *p* < 0.05) circles. Visitors at the entry (Exp β = 0.53, *p* < 0.05) and the enclosures (Exp β = 0.64, *p* < 0.05) were less likely to draw separate medium circles compared to departing visitors.

The orientations of circles on Pandas were significantly determined by gender, age, residence and visiting frequency. Males were less likely than females to orient towards the lower right (Exp β = 0.69, *p* < 0.05). Younger age groups (<18 and 18–25) were less likely to orient towards the right (Exp β = 0.95, *p* < 0.05) and lower right (Exp β = 0.46, *p* < 0.05). Sichuan locals preferred lower-right circles (Exp β = 1.22, *p* < 0.05) compared to visitors from other regions in China. First-time visitors preferred the lower-right circle (Exp β = 1.11, *p* < 0.05) to returning visitors.

Visitation stage and residence had prominent impacts on the circle sizes in panda drawings. For example, participants at the entrance and the enclosures were much more likely to draw medium—and large-sized circles compared to leaving participants, suggesting that participants toward the end of their visit tend to produce smaller representations.

In sum, demographic features such as age, visitation stage, pet ownership, gender, and visiting frequency contribute to significant changes in circle drawings with nature. For the panda images, the circle drawings were influenced by age, residence, gender, visitation stage, and visiting frequency. These findings suggest that visual representations of connection are not purely stable traits but are responsive to contextual and experiential factors within the tourism setting.

### 4.4. Associations Between Nature, Pet, and Panda Circle Drawings

Table 4 presents the Chi-square and Cramér’s V statistics used to evaluate associations between the three dimensions of circle drawings (spatial relationship, orientation, and size) across nature, pet, and panda conditions. The results indicate statistically significant associations across these domains, indicating that drawing configurations are associated across different referents.

These associations imply that individual drawing configurations in one context (e.g., nature) are related to configurations in others (e.g., pets and pandas). Notably, the strength of association is higher between pet and panda drawings than between nature–panda and nature–pet pairings, indicating a closer correspondence between these two categories. However, we also recognize that these consistencies should be interpreted with caution. It is possible that part of the observed pattern reflects individual drawing tendencies (e.g., preferences for certain sizes or spatial arrangements), rather than purely relational differences. While the inclusion of multiple dimensions (spatial relationship, orientation, and size) reduces the likelihood that the results are driven solely by stylistic factors, the present design does not fully disentangle these effects. These results demonstrate statistical association rather than causal or psychological equivalence. Therefore, the findings are interpreted as evidence of structural consistency in visual representation rather than direct indicators of underlying psychological traits.

### 4.5. Pro-Environmental Behaviours and Well-Being

This section reports statistical associations between drawing characteristics and self-reported measures. Table 5 shows that the circle sizes in Nature are related to significant changes in visitors’ recycling attitude (H = 7.335, *p* = 0.026) and willingness to participate in the giant panda conservation program (H = 8.879, *p* = 0.012). For circle drawing of the giant panda, the orientation, i.e., where participants would like to place the circle in relation to the giant panda, is associated with differences in their recycling attitude (H = 17.812, *p* = 0.023) and feeling of happiness (H = 20.258, *p* = 0.009).

Table 6 explores how circle sizes in Nature influenced pro-environmental activities, including recycling and giant panda conservation programs, through Mann–Whitney tests. Only pairs with significant differences are reported. This table shows that participants drawing medium circles exhibit significantly lower recycling intention than those drawing small circles (*p* = 0.007). In contrast, no significant difference was observed between medium-large and small-large pairs. For giant panda conservation activities, visitors drawing small circles demonstrated more significant devotion to the conservation than medium (*p* = 0.003) and large circles (0.037).

Table 7 shows that participants with circle drawings on the upper-right, lower-right, and down orientations of the panda prints were less likely to support recycling than circles on the left, up, lower left, and center. Also, upper-right, lower-right, down, and upper-left orientations are orientations that participants employed to indicate their lack of happiness compared to other orientations.

### 4.6. Visitors’ Self-Reported Level of Happiness

**Figure 10:**
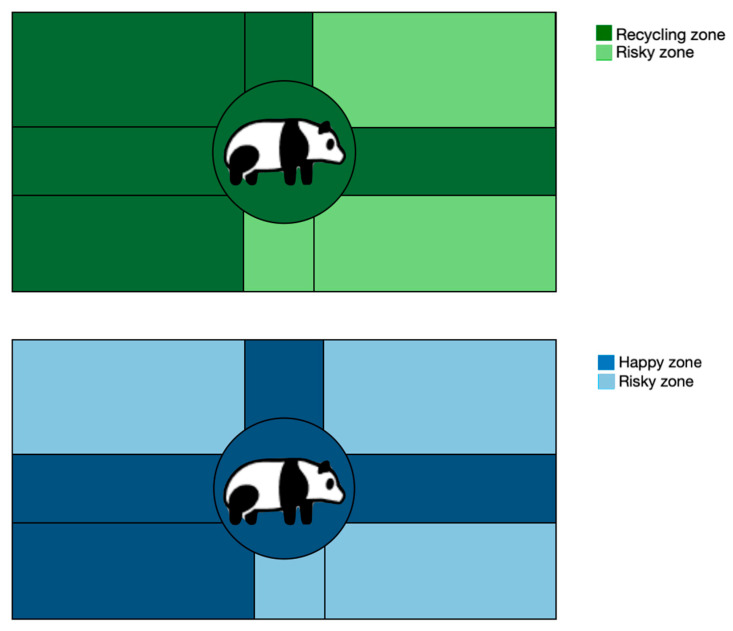
Visualizing the association between circle direction, recycling willingness and feeling of happiness with the panda print.

The top five orientations in the feeling of happiness identified in Figure 10 (Left, Lower-left, Up, Center, and Right) are categorized as “Happy zone,” while the four lower-ranked orientations (Upper-right, Lower-right, Down, Upper-left) are coded into “Risky zone.” The mean rank of “Happy zone” is 607.32 (*n* = 965), and “Risky zone” is 539.04 (*n* = 223) (U = 95,230.00, *p* = 0.000), respectively, indicating a statistically significant difference between the “Happy zone” and “Risky zone”, as visualized in Figure 10. Similarly, the green image in Figure 10 demonstrates how recycling intention is associated with the orientations of circles in the giant panda prints. Figure 10 demonstrates that upper-right, lower-right, upper-left and down are orientations associated with lower reported happiness than circles in the “Happy zone.”

While these associations are statistically significant, they should not be interpreted as strong causal relationships. These categorizations are descriptive and based on relative rankings rather than predefined psychological constructs. The behavioral and affective measures employed are simplified indicators, and the observed relationships are modest in magnitude. Rather, these findings suggest that visual representations may capture certain situational or expressive dimensions that co-vary with reported attitudes and affect in a field setting.

## 5. Discussion

One of the most intriguing questions this study attempted to answer was the particular pattern of drawn circles around the three prints. How would these circles be similar or different from each other? Was there an implicit structure that visitors followed when drawing the circles? Figure 6 shows that a large encircling circle was the most employed drawing for nature and pets, whereas the giant panda was perceived through a slightly distant, smaller circle. According to Schultz [10], the overlapping between the two circles indicates the intensity and strength of intimacy. The encircling drawings around nature and pets mean that the greatest proportion of the participants expressed a very intimate connection with them. During the fieldwork, one of the frequent explanations for the encircling drawing was “protection,” linking directly to conservation and care.

Consistent with Martin and Czellar’s [10] observation that the sizes of the circles in nature-self drawings can refer to different ideas, the bigger circle drawn around the pet over nature can, in this case, be explained with the owner’s daily engagement with, ownership of, and responsibility for their pets, topics frequently surfaced during the participant-researcher conversations. This high degree of everyday familiarity, direct interaction, and affective care may explain why emotional bonds with pets are even stronger than those with broader “nature” and significantly stronger than those with the panda.

By contrast, the separate but close circles commonly drawn around the giant panda suggest admiration from a distance—possibly shaped by the physical barriers at the Panda Base, where pandas are viewed behind glass or fencing. While the emotional appeal of the panda is strong—reflected in its global status as a flagship species—this form of connection appears mediated, symbolic, and observational rather than tactile. The visitor’s interaction with the panda is highly curated: it is regulated by zoo-like spatial arrangements, conservation narratives, and national symbolism.

As several scholars have argued [55,56], “wilderness” is not a self-evident category but a socially constructed ideal—shaped by cultural values such as solitude, purity, and separation from human society. In this light, the panda’s spatial separation in visitor drawings may reflect not only physical barriers but also a perceived ontological distance: the panda is positioned outside the realm of everyday experience—protected yet estranged. The separate circle drawn around the panda can thus be interpreted as a visual manifestation of the “designed wilderness” often associated with flagship species conservation—emotionally powerful and politically symbolic, yet psychologically distant. This presents a notable paradox for the CNI. While the index is intended to measure an individual’s felt sense of belonging to the natural world, the giant panda—precisely because of its iconic and exceptional status—may inadvertently disrupt this connection. Rather than fostering intimacy, the panda’s symbolic elevation can reinforce distance, complicating the very affective and experiential bonds that the index seeks to foster.

While Figure 6 has highlighted the most popular circles in the three prints, other patterns—especially in circle orientation—warrant closer inspection. For instance, participants’ use of “Happy zones” and “Recycling zones” (Figure 10) suggests a deliberate symbolic association between spatial placement and conservation meaning. Although this is an innovative finding, it is important to acknowledge that such patterns could also be shaped by cultural conventions in spatial representation (e.g., left-to-right movement, upper space associated with positivity) or by individual drawing styles. We recognize that this interpretation remains partly speculative, and future research should examine whether spatial symbolism is consistent across cultural groups and contexts.

Age and gender significantly influenced the changes and diversity in drawings. While pet ownership contributes to the circle drawings with nature, participants’ residence significantly impacted visitors’ relationship with the panda. More recent studies have demonstrated how age [57,58,59,60], gender [61,62,63], and residence [64,65,66] can influence people’s connectedness to nature. This study reaffirms those findings, while also showing that onsite experiences at nature-based tourism sites such as the Panda Base contribute to reshaping emotional bonds.

One field question we did not explore in this study is why participants would draw circles around nature, pets, and pandas differently. Is a panda or a pet also a part of nature, or not? This question deserves further exploration, especially because of the wide gaps in nature and panda drawing. Table 6, however, suggests that similarities in circles in the three prints are more significant and prominent, showing that the circle drawn for nature can be a strong predictor for the circle drawn for the giant panda or the pet. This finding opens possibilities for future longitudinal or cross-site studies that explore whether emotional bonds with nature transfer across categories—or remain siloed.

Finally, this study further explores Mayer and Frantz’s [3] statement that CNI can positively affect people’s well-being and pro-environmental behaviour. Despite the overall pro-environmental scores being good in this study, Table 5, Table 6 and Table 7 demonstrate that the willingness to devote to pro-environmental behaviours and feelings of happiness are highly associated with the circle drawings and their dimensions. Table 6 shows that the size of circles in nature prints can mean significant differences in pro-environmental behaviours such as recycling and giant panda conservation programs. These results show not only statistical significance, but also practical implications: larger and more intimate circle drawings are associated with stronger conservation intentions—supporting the visual and emotional mechanisms behind environmental engagement.

Additionally, the design of this study enables us to explore the orientation of the circle as a unique dimension of the circle drawing in CNI. This dimension has received less attention in previous studies, but we would like to draw more researchers’ attention to the use of orientation in further visual studies. For example, we highlight the giant panda’s “Happy zone” and “Recycling zone” in contrast to the Risky zones (Figure 10) to show how orientations have been actively employed to express feelings of happiness and willingness to recycle in this study.

## 6. Conclusions

At the heart of this research lies an examination of how visitors construct relationships with nature, animals, and specifically giant pandas within a wildlife tourism context. The findings show that visitors represent relationships with nature, pets, and giant pandas in systematically different ways, with consistent variations in spatial configuration across these referents.

This study makes two main contributions. First, methodologically, it demonstrates that visual participatory approaches can complement conventional survey-based measures by capturing relational and spatial dimensions that are not easily expressed through text-based instruments. Second, empirically, it shows that circle-drawing patterns are associated with demographic characteristics and visitation stages, indicating that these representations are shaped by both individual and situational factors.

From a practical perspective, the findings suggest that simple visual tasks such as circle drawing can be integrated into wildlife tourism settings as low-barrier tools for visitor engagement and reflection. Such approaches may support more inclusive participation, particularly among children and other groups less accessible through conventional survey methods. While the findings should be interpreted in light of the exploratory and context-specific nature of the method, they point to the potential of visual approaches for advancing research and practice in human–nature–animal relationships.

## Figures and Tables

**Table 1:** Codes and measures applied for drawing dimensions.

Dimensions	Measure of Data	Codes Assigned	Interpretation
**Spatial relationship**	Nominal	1 = Encircling 2 = Overlapping 3 = Embedded 4 = Touching 5 = Separate close 6 = Separate medium 7 = Separate distant 8 = Corner	The spatial description employed in this study is qualitative, primarily suited for manual assessments rather than precise measurements, due to limitations in resources and the large quantity of data.
**Orientation**	Nominal	0 = Center 1 = Up 2 = Upper-right 3 = Right 4 = Lower-right 5 = Down 6 = Lower-left 7 = Left 8 = Upper-left	The orientation of the circles follows a clockwise ordinal order.
**Circle size**	Ordinal	1 = Small 2 = Medium 3 = Large	The size of the circles is simplified into three stereotypes based on the comparison of the radii of the prints and the drawn circles.

**Table 2:** Demographic profiles of participants.

Variable Names and Symbols	Values (Modalities)	Code	Structure		Type of the Variables *
			*f*_i_	%	
**Age**	<18	1	121	10.2	NV
	18–25	2	282	23.7	
	26–35	3	452	38.0	
	36–45	4	115	9.7	
	46–55	5	97	8.3	
	>55	6	121	10.2	
**Gender**	Male	1	411	34.6	NV
	Female	2	777	65.4	
**Place of residence**	First-tier cities ^^^	1	171	14.4	
	Sichuan	2	209	17.6	NV
	Other	3	808	68.0	
**Visiting frequency**	First-time visitor	1	970	81.7	NV
	Returned visitor	2	218	18.3	
**Visitation stage**	Entering	1	241	20.3	NV
	At the enclosure	2	649	54.6	
	Leaving	3	298	25.1	
**Pet ownership**	Yes	1	433	36.4	NV
	No	0	755	63.6	

* Note: Acronyms in the table mean the following: NV = Nominal Variable; *f*_i_ = frequency; ^^^ Note: “First-tier cities” in China is an informal but widely used classification in urban, economic, and social research to denote the country’s most developed and globally connected metropolitan areas, typically including Beijing, Shanghai, Guangzhou, and Shenzhen. Although not an official statistical category, it is commonly employed as a pragmatic indicator of urban development level and socio-economic context in survey-based studies. “Sichuan” refers to respondents residing within Sichuan Province, where the study site is located. “Other” includes participants from all remaining regions of China outside first-tier cities and Sichuan Province, encompassing a diverse range of second- and third-tier cities as well as rural areas.

**Table 3:** Pro-environmental behaviors and wellbeing in 5-point Likert scale.

Variable Names and Symbols	Values (Modalities)	Code	Statistics	Structure		Type of the Variables *
				*f*_i_	%	
**I support recycling**		1	Mean = 4.74	5	0.4	
		2	Median = 5.00	2	0.2	
	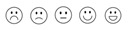	3	SD = 0.53	16	1.3	OV
		4	Skewness = −2.71	249	21.0	
		5	Kurtosis = 11.303	916	77.1	
**I want to participate in panda conservation programs**		1	Mean = 4.80	3	0.3	OV
		2	Median = 5.00	3	0.3	
	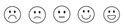	3	SD = 0.47	9	0.8	
		4	Skewness = −3.11	195	16.4	
		5	Kurtosis = 14.48	978	82.3	
**I feel happy right now**		1	Mean = 4.74	5	0.4	OV
		2	Median = 5.00	5	0.4	
	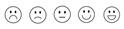	3	SD = 0.55	23	1.9	
		4	Skewness = −2.81	225	18.9	
		5	Kurtosis = 10.99	930	78.3	

* Note: Acronyms in the table mean the following: OV = Ordinal Variable; *f_i_* = frequency.

**Table 4:** Associations between dimensions of circles in paired drawings: Nature, Pet, and Panda.

			*n*	X^2^	df	Cramer’s V	*p*
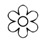	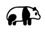	Spatial relationship		699.94	49	0.290	0.000
		Orientation	1188	399.195	64	0.205	0.000
		Circle size		376.919	4	0.398	0.000
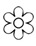	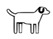	Spatial relationship		448.481	49	0.384	0.000
		Orientation	434	197.706	64	0.239	0.000
		Circle size		71.733	4	0.287	0.000
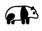	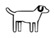	Spatial relationship		513.046	42	0.444	0.000
		Orientation	434	422.242	64	0.349	0.000
		Circle size		226.440	4	0.511	0.000

**Table 5:** Kruskal–Wallis tests for circle dimensions and pro-environmental behaviors and wellbeing.

Prints	Circle Dimensions	Recycling			Giant Panda Conservation			Feeling of Happiness		
		H	df	*p*	H	df	*p*	H	df	*p*
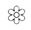	Spatial relationship	5.683	7	0.577	7.005	7	0.428	7.368	7	0.392
	Orientation	13.437	8	0.098	8.345	8	0.400	11.945	8	0.154
	Circle size	7.335	2	0.026	8.879	2	0.012	1.464	2	0.481
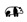	Spatial relationship	7.371	7	0.391	9.070	7	0.248	13.400	7	0.063
	Orientation	17.812	8	0.023	12.394	8	0.134	20.258	8	0.009
	Circle size	0.775	2	0.679	0.433	2	0.805	4.467	2	0.107

**Table 6:** Mann–Whitney tests for the association between NATURE circle sizes and pro-environmental behaviors.

Circle Size Pairs		*n*	Recycling				Giant Panda Conservation			
			Mean Rank	U	Z	*p*	Mean Rank	U	Z	*p*
Small-Medium	Small	368	469.43	89,818.50	−2.70	0.007	469.04	89,962.00	−2.95	0.003
	Medium	529	434.79				435.06			
Small-Large	Small	368	336.56	51,131.50	−1.42	0.155	338.61	50,374.50	−2.08	0.037
	Large	291	321.71				319.11			

**Table 7:** Kruskal–Wallis mean-ranks of recycling and wellbeing in circle direction in panda drawings.

Circle Orientation	*n*	Recycling		Feeling of Happiness	
		Mean Rank	Rank	Mean Rank	Rank
Left	39	700.63	1	664.27	1
Up	21	647.29	2	628.52	3
Lower-left	7	647.29	3	641.00	2
Center	297	604.22	4	619.82	4
Upper-left	9	601.06	5	466.83	9
Right	601	597.14	6	596.31	5
Upper-right	73	571.46	7	551.89	6
Lower-right	106	541.16	8	548.58	7
Down	35	514.14	9	501.91	8

## Data Availability

The original contributions presented in this study are included in the article. Further inquiries can be directed to the corresponding authors.

## Appendix A. Examples of Circle Sizes for Nature, Pet, and Panda-1

**Name**	**Images**	**Interpretation**
Creative art 1		The line represents the rail. The participant, as a visitor, watches the giant panda on the other side of the rail.
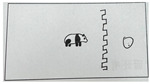
Creative art 2		The heart represent the participant’s adoration of the giant panda.
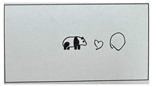
Creative art 3		The bigger circle represents a shared world where the panda and the participant co-exist.
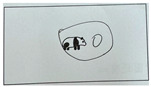
Creative art 4		The mutuality between nature and the participant.
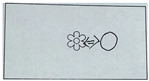
Creative art 5		The participant is in nature.
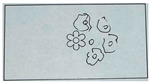
Creative 6		Chinese characters in the upper right corner, meaning “intimacy”.
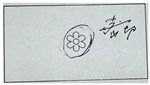
Creative 7		Loving devotion to the pet.
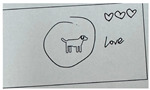
Creative 8		The participant lives together with the pet, serving the pet.
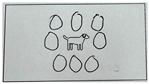
Creative 9		The owner leads the pet with a leash.
